# Does Obesity Affect Neuromuscular and Cardiovascular Adaptations after a 3-Month Combined Exercise Program in Untrained Premenopausal Middle-Aged Women?

**DOI:** 10.3390/sports11040082

**Published:** 2023-04-12

**Authors:** Konstantina Karatrantou, Vassilis Gerodimos

**Affiliations:** Department of Physical Education and Sport Science, University of Thessaly, 42100 Trikala, Greece

**Keywords:** integrated concurrent program, normal weight, aerobic dance, strength training, health, functional capacity, physical fitness, enjoyment

## Abstract

Previous studies indicated different acute adaptations between obese and lean individuals, while there is limited information with conflicting results regarding long-term adaptations. The aim of this study was to compare the efficacy of a 3-month integrated combined training between obese and lean middle-aged untrained premenopausal women. In total, 72 women (36 obese/36 lean) were divided into four groups: (a) obese exercise (OB-EG), (b) obese control (OB-CG), (c) lean exercise (L-EG), and (d) lean control (L-CG). The exercise groups followed a 3-month (3 times/week) integrated combined aerobic and strength training program. Health indices (body composition, body circumferences, blood pressure, respiratory function), functional capacity (flexibility, balance), and physical fitness (strength, aerobic capacity) were measured before and after the 3-month time period. Participants’ enjoyment was also assessed following the program. OB-EG and L-EG significantly improved (*p* < 0.05) similarly across all functional capacity and physical fitness indices (10–76%; depending on the evaluation index), except balance and strength indices of the non-preferred limb where OB-EG showed greater improvement (reducing the existing pre-training strength/balance asymmetries) than L-EG. Furthermore, both obese and lean individuals showed similarly high levels of enjoyment. This program could be effectively used in fitness settings causing similar neuromuscular and cardiovascular adaptations in obese and lean women.

## 1. Introduction

Obesity is a “multifactorial disease” characterized by disorders in the endocrine system and the metabolism and often is associated with the appearance of other chronic diseases, such as hypertension, type 2 diabetes, cardiovascular diseases, dyslipidemia, etc. [1,2,3]. Exercise has multiple positive benefits on both the physical and mental health of obese, overweight, and lean individuals [4,5,6,7,8,9,10,11,12,13,14,15]. It is important to mention, however, that the training adaptations (acute or long-term) are likely affected by obesity. In the international literature, several studies have been carried out that compared the acute responses to different modes of exercise between obese and lean individuals, while there is limited information regarding the long-term adaptations [16,17,18,19,20].

Regarding the acute responses to exercise, obese individuals (compared to lean individuals) show: (a) reduced sensitivity of β-adrenergic receptors, (b) reduced removal of perilipins, resulting in reduced activity of hormone-sensitive lipase, (c) less increased blood flow, and (d) reduced antioxidant capacity (lower levels of vitamin C, E, and β-carotene). The above disorders are associated with a reduced rate of release, transport, and delivery of fatty acids from adipose tissue to the muscle cell [19,20]. Additionally, obese individuals show changes in heart rate and blood pressure response due to autonomic nervous system dysfunction (less sympathetic and parasympathetic nervous system activation) [18,21,22] as well as a reduced rate of protein synthesis after acute resistance exercise due to reduced growth hormone concentration, reduced epinephrine release, and greater cortisol and insulin release [23,24]. The above different acute adaptations of obese individuals may also affect their long-term adaptations to aerobic and strength exercise, as previously assumed by several previous studies [6,11,16,25,26,27].

In the international literature, there are few studies that have compared the long-term effects of exercise in obese and lean individuals [6,11,16,25,26,27]. It is important to mention that (a) most of these studies assessed limited indicators of health and physical fitness, (b) some of them used a mixed sample of obese-overweight individuals influencing potentially the results of the study, and (c) these studies reported conflicting results, reporting either similar or different cardiovascular and/or neuromuscular adaptations between lean and overweight/obese individuals. More specifically, Mandroukas and colleagues [6] compared the long-term aerobic exercise adaptations (3 months) in cardiovascular and metabolic indices between obese and lean women, reporting similar responses after the end of the intervention program. On the other hand, Ciolac and Greve [16], who compared the effects of a long-term training program (12 months) between lean and overweight-obese postmenopausal women, reported that both lean and overweight/obese women improved upper and lower limb muscle strength, while cardiorespiratory fitness improved only in the lean women. Various factors such as the duration of the intervention, the exercise protocol (intensity, duration), the exercise activity, the characteristics of the sample (age, training status, mixed sample of overweight-obese individuals), etc., are likely to be responsible for the above conflicting results. Therefore, further research is needed to draw firm conclusions about the effect of obesity on long-term adaptations to exercise.

Additionally, another important aspect of this study is that it designed, implemented, and evaluated the efficacy of an integrated combined training program in obese versus lean individuals. The integrated combined training program (where the aerobic and strength routines are repeatedly altered during the training session) has lately gained popularity as an alternative exercise modality causing similar cardiovascular and neuromuscular adaptations in comparison with the serial combined training program (where the strength loading is completed prior to aerobic in each training session or vise-versa) [14,15] and reducing or eliminating the “interference effect” between strength and aerobic training [28,29,30]. Thus, the main objective of the present study was to compare the efficiency of a 3-month integrated combined strength and aerobic training program on different health (body composition, body circumferences, blood pressure, respiratory function), functional capacity (flexibility, static and dynamic balance), and physical fitness indices (strength, power, aerobic capacity) in obese (BMI > 30) versus lean (BMI < 25) middle-aged untrained premenopausal women. The participants’ enjoyment following the integrated combined training program was also examined both in obese and lean women.

## 2. Materials and Methods

### 2.1. Participants

**Figure 1:**
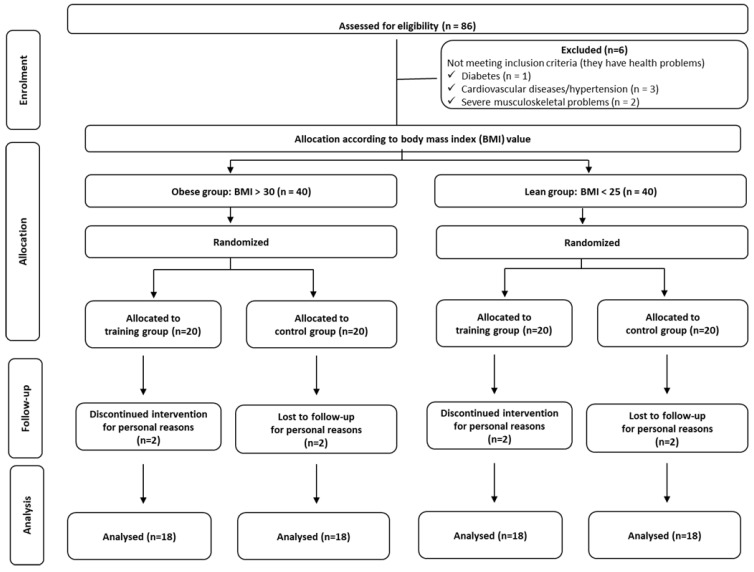
Flow chart of the progress through the phases (enrolment, intervention allocation, follow-up, and data analysis) of a randomized trial. BMI: body mass index.

Initially, 86 premenopausal middle-aged untrained women (41–51 years old) were assessed for eligibility, and six of them were excluded because they did not meet some of the inclusion criteria (they have chronic health problems). Thus, 80 women voluntarily participated in the present study and were assigned according to their body mass index (BMI) value into the obese (BMI > 30; *n* = 40) and lean groups (BMI < 25; *n* = 40). Thereafter, following baseline measurements, both obese and lean individuals were randomly assigned to either an exercise group (EG) or a control group (CG). A computer-generated list of random numbers was used for the allocation of the obese and lean individuals in one of the two groups, either exercise or control. Consequently, the participants of the present study were divided into four groups: (a) exercise group of obese women (OB-EG; *n* = 20), (b) control group of obese women (OB-CG; *n* = 20), (c) exercise group of lean women (L-EG; *n* = 20), and (d) control group of lean women (L-CG; *n* = 20). Thereafter, two participants from each group dropped out of the study because they were unable (for personal reasons) to complete either (a) the appropriate number of exercise sessions (for the OB-EG and L-EG) or (b) the post-training measurements (for the OB-CG and L-CG). Thus, the final number of participants was 72 (18 participants in each group). The flow chart of the progress, through the phases of enrolment, intervention allocation, follow-up, and data analysis, is presented in Figure 1.

Prior to the study, the women’s health status was assessed by a standardized health history questionnaire of the American College of Sports Medicine (ACSM) [31] and by a resting electrocardiogram and echocardiogram examined by a cardiologist. All subjects were (a) healthy and free of any illness, disease, or injury; (b) did not report the use of any medication; and (c) did not participate in regular physical activity for at least one year prior to the study. Before the initiation of the study, the participants were informed about the experimental procedures and possible risks and signed an informed consent form. The present study was conducted according to the Declaration of Helsinki and the ethical approval was granted by the Ethics Committee of the University of Thessaly. Age and anthropometric characteristics of the participants (per group) are presented in Table 1.

### 2.2. Study Design

First of all, a pilot study was performed to determine and finalize the testing and training procedures. After the preliminary health screening, the participants performed familiarization sessions to get accustomed to the instrumentation and the experimental testing and training procedures. Afterward, various indices of health, functional capacity, and physical fitness were assessed (pre-training measurements) on two separate days. Prior to functional capacity and physical fitness indices, the participants performed a standardized 12-min warm-up that included 5 min of low-impact aerobic dance movements and 7 min of dynamic and static stretching exercises for the whole body. During the study, the exercise groups of obese and lean women participated in a 3-month integrated combined training program, while the two control groups (OB-CG and L-CG) did not participate in any exercise program. It should be mentioned that no adverse effects or injuries were reported during the study in obese and lean middle-aged women. Two days after the completion of training, the pre-training measurements were repeated by the same investigator in the same order and at the same time of the day. The CG did not participate in any exercise program during the study at the workplace or at home. All the participants were instructed to maintain their normal daily living activities and to retain their regular dietary habits throughout the study. Furthermore, all the participants (exercise and control groups), both before and at the end of the 3-month period, completed a specific questionnaire regarding their engagement in physical activities. All the participants did not participate in regular physical activity for at least one year prior to the study, and during the study did not participate in other activities on their own.

### 2.3. Training Program

**Figure 2:**
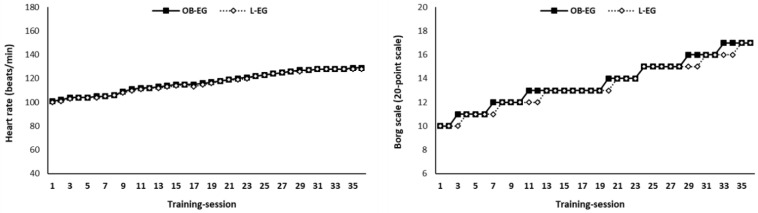
Mean heart rate and RPE in the obese exercise group (OB-EG) and lean exercise group (L-EG) throughout the 3-month training program.

The OB-EG and L-EG participated in a 3-month integrated combined training program (3 days/week; 36 training sessions in total). Each training session lasted 55–65 min and consisted of a 15 min warm-up (5 min aerobic dance, 10 min dynamic, and static stretching exercises), a 35–45 min main part of integrated combined aerobic and strength training (according to the progress of the training program), and 7 min cool-down (3 min aerobic dance and 4 min static stretching in conjunction with breathing exercises). During the main part of the training program, the aerobic and the strength training were altered in a predetermined order (3 min dance/2–2, 5 min strength exercises). The training load of aerobic and strength routines was equated between the OB-EG and L-EG. More specifically, both OB-EG and L-EG performed the same exercises during the aerobic and strength workouts using the equivalent intensity, duration, volume, and frequency of training. To ensure that the two exercise groups received similar physiological strain, we (a) performed real-time monitoring of the participants’ heart rates throughout each exercise session using the system Polar Team Solution (Science Technologies, Kempele, Finland), and (b) we assessed the rating of perceived exertion (RPE) using the 20-point Borg scale at the completion of each exercise session. The mean heart rate and RPE were not significantly different between the OB-EG and L-EG throughout the 3-month training program (Figure 2).

The main part of the training program included:

(a) Strength training: The strength workout included body weight exercises for all major muscle groups. In more detail, the program consisted of exercises for the lower (static forward lunges and step up) and upper (push-ups and dips) body as well as for abdominal and dorsal trunk muscles. The intensity during body weight exercises was modified by progressively increasing the number of repetitions (5–15 RM) and sets (2–4) during the 3-month time period, according to the recommendation of the American College of Sports Medicine [4];
(b) The aerobic training program included low-impact aerobic dance movements (i.e., forward and backward march, step touch, knee lift, heel up, kick, lateral lunges, grapevine, squats, V-step, turn step) in conjunction with continuous arm movements at the shoulder level as well as above the head. During the aerobic dance choreography, the women held a medium resistance anti-stress ball at each hand and squeezed it simultaneously according to the specific aerobic dance movements. The intensity (60–80% of the age-predicted HR_max_; 105–120 bits/min) and the duration (18–30 min) of aerobic training progressively increased during the training program according to the recommendation of the American College of Sports Medicine [4].

The gradual increase in the training load during strength (number of sets and repetitions) and aerobic (intensity as HR_max_%, rhythm, duration) workouts throughout the 3-month period is analytically presented in Table 2.

Furthermore, an indicative training session (the 6th training session) of the 3-month intervention program is analytically presented in Table 3.

### 2.4. Testing Procedures

Health, functional capacity, and physical fitness indices were measured before and after the 3-month time period. 

#### 2.4.1. Health Indices

✓ Body mass and body height were assessed using a calibrated physician’s scale (Seca model 755, Seca, Hamburg, Germany) and a telescopic height rod (Seca model 220, Seca, Hamburg, Germany), respectively, according to the recommendations of ACSM [31];
✓ Waist and hip circumferences were assessed using a conventional measuring tape (Seca 201, Seca, Hamburg, Germany), as previously described by the ACSM [31];
✓ Body composition (body fat, fat free mass) was assessed using the bioelectrical impedance method (Maltron 900) [31];
✓ Systolic and diastolic blood pressures were measured using an electronic blood pressure monitor (A & D-UA-851) [31];
✓ Forced vital capacity (FVC) and forced expiratory volume in 1 s (FEV_1_) were assessed using a portable spirometer (Micro Medical Micro), as described by the American Thoracic Society [32].

#### 2.4.2. Functional Capacity Indices

✓ Flexibility: Lower back and hamstring flexibility were measured with the sit and reach test, using a Flex-Tester box (Novel Products Inc., Rockton, IL, USA) according to the recommendations of ACSM [31]. The participants performed three maximal trials with a rest period of 10 s among trials, and the best score (in cm) was considered for analysis;
✓ Balance: The static balance was assessed on both legs using the single-limb stance tests with eyes opened and closed, as previously described by previous studies [33,34]. The participants performed three trials for each leg at each test, and the average time (in s) of the three trials was considered for analysis. Dynamic balance was also evaluated using the timed up-and-go test (TUG), as previously described by Rikli and Jones [35]. The participants performed three maximal trials with a rest period of 30 s, and the best time (in s) was used to evaluate performance.

#### 2.4.3. Physical Fitness Indices

✓ The concentric and eccentric isokinetic peak torque of knee extensors and flexors muscles were measured using a Cybex dynamometer (Cybex Norm, Ronkonkoma, NY, USA), as previously described by Tsourlou et al. [7]. The repetition with the highest moment (Nm) was used for analysis. The conventional (CON/CON and ECC/ECC) and functional (ECC/CON and CON/ECC) knee flexion (KF) to knee extension (KE) torque ratios were also calculated as previously described by Gerodimos et al. [36].
✓ Maximal and endurance handgrip strength measurements were carried out according to the recommendations of the American Society of Hand Therapists, using a portable hydraulic dynamometer (Jamar, 5030J1, Jamar Technologies, Horsham, PA, USA) as previously described by Gerodimos et al. [37];
✓ Muscular endurance of the upper body: (a) The “curl-up test” was used to measure muscular endurance of the abdominal muscles [31], (b) the “knee push-up test” and the “dip test” were used to assess muscular endurance of the chest as well as of the triceps muscles, respectively [31], and (c) the “Ito test” was used to assess muscular endurance of the trunk extensors muscles [38];
✓ Cardiorespiratory fitness was assessed using the submaximal treadmill walking test, as proposed by Ebbeling et al. [39], consisting of three 4-min stages. HR was continuously recorded using chest belt telemetry (Polar Electro, Kempele, Finland). At the end of each stage, the rating of perceived exertion (RPE) was obtained using the 20-point Borg scale. Participants’ heart rates were also measured prior to the walking test protocol at the completion of each stage and at the 1st min following the termination of the walking test.

#### 2.4.4. Enjoyment

Obese and lean participants’ enjoyment, after the integrated combined aerobic and strength training program, was assessed using the subscale of Mc Auley et al.’s intrinsic motivation questionnaire [40]. The score of each of the four questions as well as the mean overall score from all questions were used to analyze the data.

### 2.5. Statistical Analysis

All statistical analyses were performed using IBM SPSS Statistics v.26 software (IBM Corporation, Armonk, NY, USA), and the results are presented as means ± standard deviations. A statistical power analysis (software package GPower 3.0), prior to the start of the study, indicated that a total number of 72 participants (18 participants in each group) would yield adequate power (>0.85) and a level of significance (<0.05). Normal distribution was examined separately for each group using the Shapiro–Wilk test (all variables followed the normal distribution). Three-way analyses of variance (ANOVA), two BMI classifications (obese and lean) × two conditions (exercise and control) × two time-points (pre- and post-training), with repeated measures on the “time-point” factor were used to analyze the data. Sidak pairwise comparisons were applied to locate the significantly different means within and between groups. The effect sizes were calculated using the following equation: d = difference between means/pooled SD. One-way ANOVAs were used between groups to compare the relative changes from pre- to post-training in all tested parameters. Independent t-tests were used to evaluate differences in the enjoyment level between OB-EG and L-EG. Furthermore, paired t-tests were applied to examine possible differences in balance and strength measurements between the two legs (preferred vs. non-preferred leg) or the two hands (preferred vs. non-preferred hand) in each group. The significance level for all statistical analyses was set at *p* < 0.05.

## 3. Results

### 3.1. Health Indices

Analyses of variances showed significant three-way interaction effects on body composition (body mass, body fat, fat-free mass), waist and hip body circumferences, blood pressure, as well as on forced vital capacity (FVC) and forced expiratory volume in 1 s (FEV_1_) (*p* < 0.05). In more detail, in the OB-EG and L-EG body fat, waist and hip body circumferences, body mass, and blood pressure values were significantly lower at post-training versus the pre-training measurements, while FVC and FEV_1_ as well as fat-free mass values were significantly greater at post-training versus the pre-training measurements (*p* < 0.05; d = 0.80–2.0). In the OB-CG and L-CG, all the above variables did not change after the 3-month time period (*p* > 0.05) (Table 4).

Comparisons between groups revealed that all post-training values for body fat, body circumferences, body mass, and blood pressure were significantly lower in OB-EG versus OB-CG and in L-EG versus L-CG, while the fat-free mass and the FVC and FEV_1_ values were significantly higher in OB-EG versus OB-CG and in L-EG versus L-CG (*p* < 0.05). Furthermore, significant differences were also observed in post-training body fat, body circumferences, and blood pressure values between OB-EG and L-EG (*p* < 0.05). The percent changes from pre- to post-training for body fat, fat free mass, body mass, body circumferences, and blood pressure were significantly greater in OB-EG vs. L-EG (*p* < 0.05). Regarding the pre-training measurements, OB-CG and OB-CG showed higher body fat, body circumferences, and blood pressure values than the L-EG and the L-CG (*p* < 0.05).

### 3.2. Functional Capacity Indices 

Analyses of variances showed significant three-way interaction effects on all functional capacity indices (flexibility, static and dynamic balance) (*p* < 0.05). More specifically, in the OB-EG and L-EG, flexibility and static balance values were significantly higher at post-training versus the pre-training measurements, while TUG values were significantly lower at post-training versus the pre-training measurements (*p* < 0.05; d = 0.84–2.5). In the OB-CG and L-CG, all the above variables did not change after the 3-month time period (*p* > 0.05) (Table 5). 

Comparisons between groups revealed that all post-training flexibility and static balance values were significantly greater in OB-EG versus OB-CG and in L-EG versus L-CG, while TUG values were significantly lower in OB-EG versus OB-CG and in L-EG versus L-CG (*p* < 0.05). Furthermore, non-significant differences were observed in post-training functional capacity values between OB-EG and L-EG (*p* > 0.05); however, the percent changes from pre- to post-training static balance values in the non-preferred leg were significantly greater in OB-EG vs. L-EG (*p* < 0.05).

Regarding the pre-training measurements, no significant differences were observed in all functional capacity parameters among the four groups (*p* > 0.05). Nevertheless, it should be mentioned that in pre-training measurements, OB-EG and OB-CG showed significant differences in static balance between the two legs (the non-preferred leg had an approximately 13–15% lower balance than the preferred leg) (*p* < 0.05).

### 3.3. Physical Fitness Indices

#### 3.3.1. Lower Limbs Strength (Isokinetic Evaluation)

Analyses of variances showed significant three-way interaction effects on the concentric and eccentric strength of knee extensor and flexor muscles (*p* < 0.05). More specifically, in the OB-EG and L-EG, concentric and eccentric strength values of knee extensor and flexor muscles were significantly higher at post-training versus the pre-training measurements (*p* < 0.05; d = 1.5–2.8). In the OB-CG and L-CG, all the above variables did not change after the 3-month time period (*p* > 0.05) (Table 6).

Comparisons between groups revealed that all post-training concentric and eccentric strength values were significantly greater in OB-EG versus OB-CG and in L-EG versus L-CG (*p* < 0.05). Furthermore, non-significant differences were observed in post-training concentric and eccentric strength values between OB-EG and L-EG (*p* > 0.05); however, the percent changes from pre- to post-training concentric and eccentric strength values (especially of the knee flexors) in the non-preferred leg were significantly greater in OB-EG vs. L-EG (*p* < 0.05).

Regarding the pre-training measurements, no significant differences were observed in all the concentric and eccentric strength values among the four groups (*p* > 0.05). Nevertheless, it should be mentioned that in pre-training measurements, OB-EG and OB-CG showed significant differences in concentric and eccentric strength of knee flexors muscles and in muscle group ratios between the two legs (the non-preferred leg had an approximately 13–16% lower strength and lower muscle group ratios than the preferred leg) (*p* < 0.05).

#### 3.3.2. Upper Body Strength

Analyses of variances showed significant three-way interaction effects on all upper body strength tests (*p* < 0.05). More specifically, in the OB-EG and L-EG handgrip, push-ups, dip, curl-up, and Ito strength test values were significantly higher at post-training versus the pre-training measurements (*p* < 0.05; d = 0.80–5.0). In the OB-CG and L-CG, all the above variables did not change after the 3-month time period (*p* > 0.05) (Table 6).

Comparisons between groups revealed that all post-training upper body strength values were significantly greater in OB-EG versus OB-CG and in L-EG versus L-CG (*p* < 0.05). Furthermore, non-significant differences were observed in post-training upper body strength values between OB-EG and L-EG (*p* > 0.05); however, the percent changes from pre- to post-training maximal and endurance handgrip strength values in the non-preferred hand were significantly greater in OB-EG vs. L-EG (*p* < 0.05).

Regarding the pre-training measurements, no significant differences were observed in all the upper body strength values among the four groups (*p* > 0.05). Nevertheless, it should be mentioned that in pre-training measurements OB-EG and OB-CG showed significant differences in maximal and endurance handgrip strength between the two hands (the non-preferred hand had an approximately 14–17% lower strength than the preferred hand) (*p* < 0.05).

#### 3.3.3. Aerobic Capacity

Analyses of variances showed significant three-way interaction effects on heart rate and RPE values (*p* < 0.05). Specifically, in the OB-EG and L-EG, heart rate and RPE values were significantly lower at post-training versus the pre-training measurements (*p* < 0.05; d = 1.0–2.0). In the OB-CG and L-CG, the above variables did not change after the 3-month time period (*p* > 0.05).

Comparisons between groups revealed that all heart rate and RPE post-training values were significantly lower in OB-EG versus OB-CG and in L-EG versus L-CG (*p* < 0.05); however, non-significant differences were observed in post-training values between OB-EG and L-EG (*p* > 0.05). The percent changes from pre- to post-training for heart rate and RPE values were similar between OB-EG and L-EG (*p* > 0.05) and ranged from −15 to −40% depending on the measured index. Concerning the pre-training measurements, non-significant differences were observed between the four groups (*p* < 0.05). Selected aerobic capacity indices pre- and post-training are presented in Table 6.

### 3.4. Enjoyment

According to the results of the study, a greater percentage of the participants (approximately 89%) of OB-EG and L-EG reported high levels of enjoyment following the 3-month combined training program. The total mean score as well as the mean score of enjoyment at each question were similar between OB-EG and L-EG (*p* > 0.05) (Table 7).

## 4. Discussion

During the last decades, the American College of Sports Medicine has recommended a combined exercise program consisting of aerobic and strength training as the most effective approach for counteracting the detrimental effects of a sedentary lifestyle and reducing the prevalence of health risk factors in obese, overweight, and lean individuals [4]. For this reason, sports and health professionals all over the world design and implement different serial and integrated combined exercise programs using various activities, demonstrating promising results in different health, functional capacity, and physical fitness parameters [5,6,7,8,9,10,11,12,13,14,15,41,42]. The present study designed and implemented, with success (without adverse effects) in both obese and lean individuals, a supervised integrated combined exercise program including aerobic and strength training, assessing a comprehensive health, functional capacity, and physical fitness profile. The main finding of this study is that a 3-month supervised combined exercise program induces similar cardiovascular and neuromuscular adaptations in obese and lean middle-aged untrained women.

More specifically, this study indicated that a 3-month (3 days/week) integrated combined aerobic (using low-impact aerobic dance movements) and strength training program (using body weight exercises for the whole body) induced significant improvements in different neuromuscular indices (flexibility, balance, lower and upper body strength) for both obese and lean untrained middle-aged individuals. Previous studies that implemented different combined exercise programs also reported significant improvements in neuromuscular indices both in obese and lean individuals, while others failed to observe significant training adaptations [7,8,10,11,14,15,41,43]. The equivocal results among studies regarding neuromuscular responses may be attributed to differences in subjects’ characteristics, loading parameters, exercise modalities, as well as to the order of exercises. For example, several studies indicated that the residual fatigue caused by prior endurance training reduces the neural input to the endurance-exercised muscle causing decrements in force output and rate of force development and attenuating neuromuscular adaptations [44]. On the other hand, other studies showed that the mode of combined training (serial or integrated) and the order of exercises has no effect on the development of muscle strength and power [45]. The integrated combined training mode that we used in the present study may reduce or eliminate the “interference effect” between strength and aerobic training due to less muscle soreness and faster muscle recovery following exercise [28,29,30].

One of the most important findings of this study is that, before the start of the program, obese women had significant strength and balance asymmetries (13–17%) between the two limbs (the preferred limb prevailed against the non-preferred limb) in all measured parameters (concentric and eccentric isokinetic peak torque of the knee joint, static balance with eyes opened and closed, maximal and endurance handgrip strength), increasing the incidence of injuries. Although there were some findings in the literature regarding the greater imbalances, asymmetries, and instabilities of obese individuals compared to lean individuals [46,47,48,49,50], this research strengthens the findings of those studies evaluating asymmetries in various tested parameters. It should also be mentioned that following the exercise program, obese women reported greater percentage improvements compared to lean women on (a) static balance of the non-preferred leg, (b) concentric and eccentric isokinetic strength of knee flexor muscles as well as on conventional and functional muscle group torque ratios of the non-preferred leg, and (c) maximal and endurance handgrip strength of the non-preferred hand. To the best of our knowledge, this is the first study that compared bilateral differences in strength and balance training adaptations between obese and lean individuals. In this study, the presence of asymmetries between the two legs or the two hands (differences above 10%) as well as the lower muscle group torque ratios in the pre-training measurements of obese women may account for greater improvements in the balance and strength indices of the non-preferred leg. The strength and balance asymmetries observed in this study in obese women were significantly reduced following the 3-month training program and returned to normal levels, thus reducing the possibility of muscle injuries. Although, as we mentioned above, no previous research has compared bilateral differences in strength and balance training adaptations between obese and lean individuals, there are some previous findings that support the results of the present study. Specifically, a previous study demonstrated that obese individuals compared to non-obese individuals have lower knee flexor values and lower muscle group torque ratios [46]. The authors believe that the knee flexor muscles of obese individuals might not be able to effectively counteract the action of the knee extensor during extension movements [46]. From the results of this and the aforementioned studies, it seems that it is very important to design and implement multilateral exercise programs emphasizing the uniform development of both agonist and antagonist muscles as well as the uniform development of both sides of the body, avoiding imbalances and asymmetries between the two legs and the two hands. However, future studies could further examine and elucidate in more detail the presence and treatment of limb asymmetries in obese individuals.

Additionally, an important finding of this study is that the integrated combined exercise program decreased body fat, body circumferences, and blood pressure and increased respiratory function both in obese and lean middle-aged women. The reduction of body fat, body circumferences, and blood pressure was greater in obese women, which showed higher values before the start of the program, than in lean middle-aged women. The results of this study are in accordance with previous studies that investigated the effects of different exercise programs and reported greater alterations in health indicators of obese women [6,11,16,25,26,27]. However, the total amount of improvement revealed in this study in several indicators (i.e., body fat) is different than in some other studies. The divergence in the total amount of improvement of ours and the previous studies may be correlated with different independent and/or interactive factors such as the type of intervention (exercise, nutrition, mixed exercise and nutrition), the training contents (strength training, aerobic training and combined strength and aerobic training), the training load (i.e., training frequency) of the program, and/or the subject’s characteristics (physical fitness level). Furthermore, the results of the present study reveal significant similar cardiovascular adaptations following the combined exercise program both in obese and lean individuals, as previously described and by other studies [6,11]. In contrast, other studies reported impaired cardiovascular responses in obese/overweight versus lean individuals [16].

Beyond the efficacy, an exercise program should be safe for individuals. The control of safety and the continuous monitoring of training are important aspects for the successful implementation of an exercise program, especially in untrained and obese individuals who show an increased risk of injuries [46] and, as a result, a greater incidence of dropout due to adverse effects. The program of the present study was specifically designed and adjusted to the participants’ possibilities, consisting of (a) low-impact aerobic dance movements that do not strain the muscle joints and are considered safe for untrained and obese individuals and (b) body weight strength exercises that do not require extra familiarization with external resistances. Furthermore, an important advantage of this study is the continuous monitoring of training (recording of heart rate and rating of perceived exertion—RPE) throughout the 3-month period, where no differences were observed between lean and obese individuals.

Finally, the results of the present study show that a great percentage of both obese and lean middle-aged women were absolutely satisfied with their participation in the integrated combined strength and aerobic training program. In the literature, none of the studies that compare training adaptations between obese and lean individuals evaluate the participants’ enjoyment following the program. The evaluation of enjoyment following an exercise program is of crucial importance, especially in population groups with reduced exercise adherence and increased percentage of dropout such as obese, elderly, and untrained individuals. Previous studies [8,10,15,51,52,53], in accordance with the results of the present study, showed high levels of enjoyment following different exercise interventions in various populations.

The present study has some limitations that could affect its outcomes and, as a result, their generalization. Firstly, the findings of this study are limited to the use of a 3-month combined exercise program consisting of aerobic training using low-impact aerobic dance movements and strength training using body weight exercises. Future studies could investigate possible differences in training adaptations between obese and lean individuals using other exercise modalities and training interventions of greater duration (above 3 months). Furthermore, our findings are clearly limited to untrained middle-aged premenopausal obese (with no other accompanying chronic diseases) and lean women. Future studies should examine the efficacy and safety of integrated combined training programs in obese individuals with chronic diseases, in individuals of other age groups (i.e., young adults or elderly), in individuals with different training statuses (physically active-trained individuals), as well as in women at different menopause stages (menopause, post-menopause). Finally, another limitation of this study is the method used for the evaluation of body composition (bioelectrical impedance method without specialized equation according to obesity level) both in lean and obese individuals before and after the 3-month period. Although the bioelectrical impedance method is commonly used for body composition assessment as a simple, non-invasive, and low-cost method that estimates the total body water (TBW) through the resistance of the body, it has some limitations that can affect the results of the study regarding body composition especially in obese individuals (due to the fact that a) most predictive equations have been developed in normal-weight subjects, and b) body water distribution may be different in obese individuals). These limitations of the bioelectrical impedance method could overestimate fat-free mass and underestimate fat mass, especially in obese individuals, compared to a gold standard method such as dual-energy X-ray absorptiometry (DXA).

## 5. Conclusions

In conclusion, a 3-month integrated combined exercise program consisting of aerobic (low impact aerobic dance movements) and strength (body weight exercise for the whole body) workouts is an effective and enjoyable intervention causing similar cardiovascular and neuromuscular adaptations in obese and lean middle-aged untrained women. An important finding of this study is that, before the start of the program, obese women had significant strength and balance asymmetries between the two limbs in all measured parameters (concentric and eccentric isokinetic peak torque of the knee joint, static balance with eyes opened and closed, maximal and endurance handgrip strength). These strength and balance asymmetries, both in the lower and upper limbs, were significantly reduced in obese women following the 3-month training program, returning to normal levels.

## Figures and Tables

**Table 1:** Age and anthropometric characteristics of the sample (mean ± standard deviation).

Characteristics	OB-EG (*n* = 18)	OB-CG (*n* = 18)	L-EG (*n* = 18)	L-CG (*n* = 18)
Age (years old)	45.97 ± 4.43	46.00 ± 4.35	46.09 ± 4.43	45.99 ± 4.20
Body mass (kg)	89.19 ± 4.44	88.85 ± 3.35	60.91 ± 3.56	61.00 ± 3.50
Body height (m)	1.61 ± 0.06	1.62 ± 0.05	1.64 ± 0.06	1.63 ± 0.07
BMI (kg/m^2^) *	34.43 ± 2.35	33.91 ± 2.15	22.64 ± 1.33	22.93 ± 1.25

* BMI: body mass index = body mass/body height^2^. OB-EG: exercise group of obese women, OB-CG: control group of obese women, L-EG: exercise group of lean women, L-CG: control group of lean women.

**Table 2:** Gradual increase of the training load during strength and aerobic workouts.

Weeks												
	1	2	3	4	5	6	7	8	9	10	11	12
**Aerobic training (low-impact aerobic dance)**												
Intensity (HR_max_%)	60–65	65–70	65–70	65–70	70–75	70–75	70–75	70–75	70–75	75–80	75–80	75–80
Rhythm (bpm)	105	110	110	110	110	115	115	115–118	115–118	115–118	118–120	118–120
Set × duration (min)	6 × 3	6 × 3	7 × 3	7 × 3	8 × 3	8 × 3	8 × 3	9 × 3	9 × 3	9 × 3	10 × 3	10 × 3
Total duration (min)	18	18	21	21	24	24	24	27	27	27	30	30
**Strength training (body weight exercises)**												
Set												
Lunges	2	2	2	2	3	3	3	3	3	4	4	4
Step ups	2	2	2	2	3	3	3	3	3	4	4	4
Abdominals	2	2	2	2	3	3	3	3	3	4	4	4
Dorsals	2	2	2	2	3	3	3	3	3	3	4	4
Push-ups	2	2	2	2	3	3	3	3	3	4	4	4
Dips	2	2	2	2	3	3	3	3	3	4	4	4
Repetitions												
Lunges	8	8	8	10	10	10	10	10	12	12	12	12
Step ups	8	8	8	10	10	10	10	10	12	12	12	12
Abdominals	10	10	10	12	12	12	12	15	15	15	15	15
Dorsals	10	10	10	12	12	12	12	15	15	15	15	15
Push-ups	6–8	6–8	8–10	8–10	10	10	10	10	10	12	12	12
Dips	5–6	5–6	5–6	6–8	6–8	6–8	7–9	7–9	7–9	8–10	8–10	8–10

% HRmax: percentage of the age-predicted maximum heart rate as recorded during the training program by the Polar Team Solution System.

**Table 3:** An indicative training session (6th training session) of the 3-month combined exercise program.

Total Duration of the 6th Training Session: 60 Min	
**Warm-up** **(15 min)**	Low-impact aerobic dance movements (5 min).
	Dynamic and static stretching exercises for the whole body (10 exercises; 2 sets × 15 s for static/10 reps for dynamic).
**Main part** **(38 min)**	**Block 1.** Aerobic dance (3 min)/2 strength exercises: (a) dorsals (1 set × 12 reps), (b) knee push-ups (1 set × 7 reps).
	**Block 2.** Aerobic dance (3 min)/2 strength exercises: (a) dorsals (1 set × 12 reps), (b) knee push-ups (1 set × 7 reps).
	**Block 3.** Aerobic dance (3 min)/1 strength exercise: lunges (1 set × 10 reps per leg).
	**Block 4.** Aerobic dance (3 min)/1 strength exercise: lunges (1 set × 10 reps per leg).
	**Block 5.** Aerobic dance (3 min)/2 strength exercises: (a) sit-ups (1 set × 12 reps), (b) dips (1 set × 7 reps).
	**Block 6.** Aerobic dance (3 min)/2 strength exercises: (a) sit-ups (1 set × 12 reps), (b) dips (1 set × 7 reps).
**Cool down** **(7 min)**	Low-impact aerobic dance movements (3 min).
	Static stretching for the whole body in conjunction with breathing exercises (4 min).

**Table 4:** Health indices per group and measurement in middle-aged premenopausal women (means ± SD).

Variables	Measurement	OB-EG	OB-CG	L-EG	L-CG
Body mass (kg)	Pre Post	89.2 ± 4.4 84.0 ± 3.6 *#	88.9 ± 3.4 89.1 ± 3.5	60.9 ± 3.6 58.5 ± 3.3 *#^†^	61.0 ± 3.5 61.5 ± 3.3
Body fat (%)	Pre Post	48.5 ± 3.3 41.0 ± 3.6 *#	47.6 ± 2.3 47.9 ± 3.6	29.3 ± 2.3 27.0 ± 3.3 *#^†^	29.0 ± 3.5 29.5 ± 3.3
Fat-free mass (kg)	Pre Post	45.9 ± 3.4 49.6 ± 3.6 *#	46.5 ± 2.6 46.4 ± 3.6	43.1 ± 2.3 44.8 ± 3.5 *#^†^	43.3 ± 3.6 43.9 ± 3.4
Hip circumference (cm)	Pre Post	118.9 ± 8.7 111.9 ± 8.8 *#	117.3 ± 6.5 118.3 ± 7.8	97.9 ± 5.2 95.0 ± 5.5 *#^†^	98.1 ± 4.2 98.4 ± 4.5
Waist circumference (cm)	Pre Post	106.3 ± 8.5 98.2 ± 8.3 *#	107.0 ± 7.5 107.5 ± 8.2	84.5 ± 5.1 80.7 ± 5.8 *#^†^	85.5 ± 4.1 846 ± 4.8
Systolic blood pressure (mmHg)	Pre Post	118.0 ± 10.0 110.1 ± 8.0 *#	118.0 ± 10.0 120.0 ± 9.5	104.9 ± 8.0 100.6 ± 6.7 *#^†^	105.6 ± 8.0 104.9 ± 8.7
Diastolic blood pressure (mmHg)	Pre Post	84.4 ± 8.9 76.9 ± 9.2 *#	85.5 ± 7.9 84.9 ± 8.2	70.8 ± 4.8 67.2 ± 3.8 *#^†^	71.0 ± 3.8 72.2 ± 2.8
Forced vital capacity (L)	Pre Post	3.17 ± 0.6 3.32 ± 0.5 *#	3.19 ± 0.4 3.20 ± 0.6	3.21 ± 0.3 3.35 ± 0.4 *#	3.20 ± 0.5 3.22 ± 0.4
Forced expiratory volume in 1 s (L)	Pre Post	2.63 ± 0.2 2.73 ± 0.4 *#	2.65 ± 0.3 2.64 ± 0.2	2.63 ± 0.3 2.75 ± 0.3 *#	2.64 ± 0.2 2.63 ± 0.4

Where * *p* < 0.05 refers to a statistically significant difference between pre- and post-intervention programs in OB-EG and L-EG, # *p* < 0.05 refers to a statistically significant difference between OB-EG and OB-CG and between L-EG and L-CG in the post-training measurement, and ^†^ *p* < 0.05 refers to a statistically significant difference between OB-EG and L-EG in post-training measurements. OB-EG: exercise group of obese women, OB-CG: control group of obese women, L-EG: exercise group of lean women, L-CG: control group of lean women.

**Table 5:** Functional capacity indices per group and measurement in middle-aged premenopausal women (means ± SD).

Variables	Measurement	OB-EG	OB-CG	L-EG	L-CG
Flexibility Sit and reach test (cm)	Pre Post	24.2 ± 7.2 30.1 ± 5.2 *#	25.2 ± 6.5 25.5 ± 5.6	26.2 ± 8.3 32.4 ± 6.3 *#	25.9 ± 8.5 26.4 ± 8.3
Static balance-opened eyes (s) Preferred leg	Pre Post	41.8 ± 8.0 53.7 ± 7.7 *#	41.7 ± 8.1 41.9 ± 7.6	41.9 ± 8.2 55.2 ± 7.7 *#	42.0 ± 8.5 42.1 ± 8.7
Non-preferred leg	Pre Post	37.3 ± 7.4 ^†^ 52.7 ± 7.7 *#	37.4 ± 7.4 ^†^ 38.0 ± 7.7	40.8 ± 7.9 56.3 ± 8.7 *#	41.0 ± 7.5 41.9 ± 7.7
Static balance-closed eyes (s) Preferred leg	Pre Post	11.0 ± 5.0 20.0 ± 9.2 *#	11.3 ± 6.1 12.0 ± 7.6	12.0 ± 5.9 20.5 ± 8.6 *#	11.6 ± 6.5 11.0 ± 6.7
Non-preferred leg	Pre Post	9.0 ± 5.1 ^†^ 19.6 ± 8.5 *#	9.4 ± 6.4 ^†^ 9.6 ± 7.7	11.0 ± 5.8 20.6 ± 9.0 *#	10.8 ± 5.5 11.3 ± 5.7
Dynamic balance TUG test (s)	Pre Post	4.38 ± 0.5 3.88 ± 0.5 *#	4.36 ± 0.4 4.35 ± 0.5	4.27 ± 0.4 3.88 ± 0.5 *#	4.28 ± 0.3 4.26 ± 0.5

Where * *p* < 0.05 refers to a statistically significant difference between pre- and post-intervention programs in OB-EG and L-EG, # *p* < 0.05 refers to a statistically significant difference between OB-EG and OB-CG and between L-EG and L-CG in the post-training measurement, and ^†^ *p* < 0.05 refers to a statistically significant difference in pre-training measurement between the two legs in OB-EG and OB-CG in post-training measurements. OB-EG: exercise group of obese women, OB-CG: control group of obese women, L-EG: exercise group of lean women, L-CG: control group of lean women.

**Table 6:** Physical fitness indices per group and measurement in middle-aged premenopausal women (means ± SD).

Variables	Measurement	OB-EG	OB-CG	L-EG	L-CG
**Lower Body Strength**					
Concentric peak torque (Nm)					
Extensors PL	Pre Post	113.0 ± 21.8 142.8 ± 19.3 *#	112.9 ± 23.2 115.6 ± 20.0	111.0 ± 17.8 136.0 ± 15.6 *#	109.9 ± 22.2 110.6 ± 20.7
Extensors NPL	Pre Post	114.4 ± 26.4 142.6 ± 21.5 *#	113.0 ± 22.8 112.8 ± 20.5	111.6 ± 18.0 135.0 ± 19.5 *#	110.4 ± 22.8 112.9 ± 18.5
Flexors PL	Pre Post	69.7 ± 12.7 89.2 ± 10.4 *#	69.1 ± 11.9 70.0 ± 11.7	68.7 ± 10.2 84.2 ± 10.5 *#	68.9 ± 10.9 69.2 ± 11.7
Flexors NPL	Pre Post	60.0 ± 12.8 ^†^ 89.5 ± 10.6 *#	59.9 ± 12.5 ^†^ 60.0 ± 13.7	69.1 ± 14.1 82.5 ± 10.4 *#	67.8 ± 14.9 67.5 ± 14.5
Eccentric peak torque (Nm)					
Extensors PL	Pre Post	165.0 ± 34.7 194.0 ± 30.7 *#	162.0 ± 34.7 160.0 ± 31.7	158.0 ± 35.3 185.7 ± 34.3 *#	159.5 ± 34.1 161.2 ± 36.3
Extensors NPL	Pre Post	166.7 ± 36.7 193.0 ± 37.3 *#	163.7 ± 32.8 162.6 ± 37.7	155.7 ± 37.3 182.3 ± 33.3 *#	156.0 ± 37.3 157.0 ± 38.3
Flexors PL	Pre Post	97.6 ± 13.9 116.5 ± 14.0 *#	94.8 ± 16.8 93.6 ± 14.3	92.7 ± 16.3 112.6 ± 12.8 *#	93.3 ± 15.4 95.8 ± 15.6
Flexors NPL	Pre Post	86.0 ± 16.7 ^†^ 116.2 ± 19.5 *#	83.5 ± 19.7 ^†^ 83.0 ± 18.6	91.7 ± 11.8 112.5 ± 16.3 *#	92.4 ± 12.1 93.6 ± 14.3
**Upper body strength**					
Maximal handgrip strength (kg)					
Preferred hand	Pre Post	32.7 ± 5.9 36.6 ± 4.9 *#	32.5 ± 5.8 32.6 ± 6.0	31.3 ± 5.9 34.9 ± 5.5 *#	31.6 ± 5.7 32.0 ± 6.1
Non-preferred hand	Pre Post	29.0 ± 4.1 ^†^ 36.0 ± 4.5 *#	28.5 ± 5.9 ^†^ 28.4 ± 5.7	31.3 ± 5.8 34.8 ± 5.3 *#	31.0 ± 6.1 30.7 ± 6.2
Endurance handgrip strength (time in s)					
Preferred hand	Pre Post	62.4 ± 17.0 92.1 ± 25.5 *#	62.6 ± 20.0 61.5 ± 23.7	62.3 ± 20.8 91.1 ± 26.6 *#	62.4 ± 22.0 61.6 ± 23.7
Non-preferred hand	Pre Post	53.0 ± 20.0 ^†^ 89.9 ± 23.7 *#	52.6 ± 21.8 ^†^ 52.0 ± 19.7	58.0 ± 23.3 86.6 ± 23.0 *#	57.6 ± 23.8 58.3 ± 22.7
Sit up (reps)	Pre Post	8.6 ± 4.0 16.9 ± 2.7 *#	8.5 ± 3.5 8.7 ± 3.7	9.6 ± 3.3 18.1 ± 2.2 *#	9.0 ± 3.8 8.9 ± 3.7
Ito test (s)	Pre Post	61.0 ± 22.2 122.4 ± 25.7 *#	63.4 ± 26.0 62.7 ± 23.0	65.2 ± 20.0 126.6 ± 22.3 *#	64.6 ± 23.8 63.7 ± 24.7
Push-ups (reps)	Pre Post	3.4 ± 1.4 12.7 ± 3.5 *#	3.5 ± 1.3 3. 7 ± 1.2	3.6 ± 1.2 14.5 ± 4.0 *#	3.7 ± 1.2 3.8 ± 1.3
Dip test (reps)	Pre Post	2.8 ± 1.5 11.0 ± 2.9 *#	2.9 ± 1.2 2.7 ± 1.0	3.1 ± 1.0 13.0 ± 3.9 *#	3.0 ± 1.3 2.9 ± 1.4
**Aerobic capacity**					
HR_test_ (beats/min)	Pre Post	139.8 ± 12.7 119.4 ± 10.4 *#	140.6 ± 13.4 141.7 ± 13.7	141.1 ± 11.4 120.4 ± 10.9 *#	140.4 ± 12.4 139.7 ± 13.0
RPE_test_	Pre Post	12.8 ± 2.0 9.6 ± 1.9 *#	12.6 ± 1.8 13.0 ± 2.7	12.5 ± 1.5 9.4 ± 1.7 *#	12.6 ± 1.4 12.7 ± 1.2
HR_rec_ (beats/min)	Pre Post	119.6 ± 15.8 99. 7 ± 14.7 *#	118.6 ± 13.4 117. 7 ± 13. 7	117.1 ± 16.0 96.5 ± 15.4 *#	118.6 ± 14.8 119.7 ± 13.3

Where * *p* < 0.05 refers to a statistically significant difference between pre and post-intervention programs in OB-EG and L-EG, # *p* < 0.05 refers to a statistically significant difference between OB-EG and OB-CG and between L-EG and L-CG in the post-training measurement, and ^†^ *p* < 0.05 refers to a statistically significant difference in pre-training measurement between the two legs or the two hands in OB-EG and OB-CG in post-training measurements. OB-EG: exercise group of obese women, OB-CG: control group of obese women, L-EG: exercise group of lean women, L-CG: control group of lean women, PL: preferred leg, NPL: non-preferred leg, HR_test_: heart rate values at the end of the second stage of the submaximal test, HR_rec_: heart rate values the 1 min following the submaximal test, RPE_test_: rating of perceived exertion at the end of the second stage of the submaximal test.

**Table 7:** Enjoyment values in obese and lean exercise groups following the 3-month combined training program.

	OB-EG	L-EG
Question 1 (score)	4.5 ± 0.3	4.6 ± 0.2
Question 2 (score)	4.7 ± 0.2	4.7 ± 0.3
Question 3 (score)	4.2 ± 0.3	4.3 ± 0.5
Question 4 (score)	4.3 ± 0.4	4.2 ± 0.3
Total score from all questions	4.4 ± 0.3	4.5 ± 0.4

OB-EG: exercise group of obese women, L-EG: exercise group of lean women.

## Data Availability

Data are unavailable due to privacy or ethical restrictions.
